# The Representation of Three-Dimensional Space in Fish

**DOI:** 10.3389/fnbeh.2016.00040

**Published:** 2016-03-08

**Authors:** Theresa Burt de Perera, Robert I. Holbrook, Victoria Davis

**Affiliations:** ^1^Department of Zoology, University of OxfordOxford, UK; ^2^School of Computing, University of LeedsLeeds, UK

**Keywords:** navigation, spatial cognition, fish, hippocampal place cells, cognitive map

## Abstract

In mammals, the so-called “seat of the cognitive map” is located in place cells within the hippocampus. Recent work suggests that the shape of place cell fields might be defined by the animals’ natural movement; in rats the fields appear to be laterally compressed (meaning that the spatial map of the animal is more highly resolved in the horizontal dimensions than in the vertical), whereas the place cell fields of bats are statistically spherical (which should result in a spatial map that is equally resolved in all three dimensions). It follows that navigational error should be equal in the horizontal and vertical dimensions in animals that travel freely through volumes, whereas in surface-bound animals would demonstrate greater vertical error. Here, we describe behavioral experiments on pelagic fish in which we investigated the way that fish encode three-dimensional space and we make inferences about the underlying processing. Our work suggests that fish, like mammals, have a higher order representation of space that assembles incoming sensory information into a neural unit that can be used to determine position and heading in three-dimensions. Further, our results are consistent with this representation being encoded isotropically, as would be expected for animals that move freely through volumes. Definitive evidence for spherical place fields in fish will not only reveal the neural correlates of space to be a deep seated vertebrate trait, but will also help address the questions of the degree to which environment spatial ecology has shaped cognitive processes and their underlying neural mechanisms.

For decades, research has focussed on elucidating how animals navigate across familiar terrain. In vertebrates, an efficient system involves learning and remembering incoming sensory information and encoding this into a representation that contains current location and heading direction (O’Keefe and Nadel, 1978). With this information, an animal can work out where it is, and in which direction it should proceed in order to reach a goal. The neural basis of this representation has been intensively studied in rodents and involves a number of interconnected brain structures that encode both distance and direction.

Hippocampal place cells were first described by O’Keefe and Dostrovsky (1971). The firing rates of individual cells increase when a rat is located in one particular area of its environment, and together, these cells seem to operate as a neural map. Further research has revealed that metric information is encoded within a population of place cells; that is, distance traveled and direction (Hartley et al., 2000). But from where is this information conveyed? The answer lies in head direction and grid cells, which are located in the entorhinal cortex and are afferent to place cells. The former encode the direction in which the animal is facing; each cell exhibiting maximum firing rates when an animal faces a given direction (Taube et al., 1990). Visual cues act as a frame of reference, stabilizing and calibrating the directional signal in the environment (Goodridge et al., 1998) and inputs from self-motion update the signal as the animal travels. Grid cells appear to encode the distance that an animal has traveled (Hafting et al., 2005). Populations of these cells fire in a tessellating hexagonal grid-like pattern over a range of scales, and seem to measure distance via self-motion cues, operating in a similar way to graph paper. These interconnecting brain structures are thought to operate together to form a map of local space that the animal can use to navigate efficiently.

Most studies, both behavioral and neurophysiological, have considered how animals navigate in two-dimensions. However, the real world is three-dimensional and this third dimension varies in complexity depending on the environment. Even surface-bound animals will usually be subjected to undulating terrain, but this is taken to an extreme in unbound animals, which move freely through volumetric environments such as fish (Holbrook and Burt de Perera, 2009, 2011b, 2013) and birds (Flores-Abreu et al., 2014). Current discussion is centered on how vertebrate animals encode three-dimensional space and in particular, whether there are differences between the vertical and horizontal dimensions (Jeffery et al., 2015). Such encoding differences could potentially result in variation in the precision of spatial memory between the horizontal plane and the vertical axis and through ontogeny and/or evolution, this might vary between surface traveling animals and those that fly or swim freely through volumes (Holbrook and Burt de Perera, 2013; Flores-Abreu et al., 2014).

Rats (*Rattus norvegicus*) are surface-bound, although they are able to climb through three-dimensional space. Rats exploring a cubic lattice maze navigated by a layer strategy, searching each horizontal level before moving vertically to explore the next level (Jovalekic et al., 2011). However, despite the bias towards making horizontal movements during exploratory tasks, in spatial memory tasks, rats appear to learn the vertical location of a reward before the horizontal (Grobéty and Schenk, 1992). Jovalekic et al. (2011), argued that this might be as a result of its energetic cost although, the energetic cost hypothesis was not consistent with other studies (Flores-Abreu et al., 2014). In a goal oriented task in a lattice maze, Flores-Abreu et al. (2014) also showed that rats learned the horizontal component more accurately than the vertical, despite having made more vertical than horizontal movements. This is consistent with the rats prioritizing learning of the vertical component first, and only once this learning is complete, moving onto the horizontal.

This behavioral evidence suggests that in surface-bound rats, both the horizontal and vertical components of space are learned, but used separately with a bias towards a use of the horizontal. What about the neural basis of the rats’ representation of space? In a foraging task on a vertical pegboard, Hayman et al. (2011) found that place cell fields were “stretched” in the vertical axis compared to recordings from the same cells when the animals were traveling horizontally. As suggested by Ulanovsky (2011), a possible reason is that the elongation of place cells reflected the repetitive structure of the pegboard environment. When trained in a repetitive horizontal maze, place fields formed elongated stripes, which were likely to be an artifact of the experimental set up (Singer et al., 2010). However, Hayman et al. (2011) argue that the vertical dimension is encoded with greater error than the horizontal, either because of their ontogeny (rats spend more time walking across horizontal surfaces than vertical), or because the place fields in the animals have evolved to have a particular form.

Putting these results together (and given the challenges of coding three-dimensional space that are related to its geometric properties), Jeffery et al. (2015), have surmised that surface-bound animals encode three-dimensional space as a mosaic of two-dimensional maps that are locally planar, but related to each other metrically. In other words, the suggestion is that volumetric space is encoded anisotropically (differently in vertical and horizontal dimensions). However, this hypothesis is not without its critics, the main problem being that Jeffery’s rats were raised in small, vertically constrained boxes. As Jeffery et al. (2015) themselves point out, this reduction in vertical complexity might be the reason why the neural representation has less resolution in the vertical than in the horizontal dimension. While a likely explanation is that volumetric space is encoded anisotropically in surface-bound animals due to the their spatial ecology, this has not been shown unequivocally.

Recent work from free-flying Egyptian fruit bats (*Rousettus aegyptiacus*) has produced intriguing data. When flying through a volume, the place fields from different neurons were not compressed or elongated in either dimension, unlike in the rats (Yartsev and Ulanovsky, 2013). In other words, the vertical and horizontal components of space appear to be coded with equal error. This isotropic representation of three-dimensional space would make adaptive sense for animals that fly freely through volumes, allowing equally precise navigation in both dimensions.

Like bats, hummingbirds (*Selasphorus rufus*), are not surface-bound, and in experiments moved equally in all three-dimensions when tested in a cubic maze with a rewarded location. The expectation might then be that the vertical component of space is more important to these animals than those that are surface-bound, and indeed in a lattice structure they were able to locate a food reward in the vertical dimension more accurately than in the horizontal (Flores-Abreu et al., 2014). This is consistent with a previous study in which the hummingbirds were also more accurate at remembering the location of a reward in the vertical axis than the horizontal dimension (Hurly et al., 2010). However, the picture is not entirely clear-cut as in a separate study, birds were trained to locate a rewarded flower more accurately in the horizontal than the vertical (Flores-Abreu et al., 2013). The presence of salient visual horizontal landmarks and no equivalent vertical landmarks or other cues is likely to explain this latter result.

The current evidence suggests that how an animal moves through its environment determines the accuracy of horizontal and vertical memory in three-dimensional spatial tasks; specifically whether it is able to freely move through three-dimensions with six degrees of freedom [three translational movements (forwards/backwards, left/right, up/down) and three rotational (pitch, roll and yaw)] or is surface-bound and restricted to three degrees of freedom (Holbrook and Burt de Perera, 2009; Burt de Perera et al., 2013; Flores-Abreu et al., 2014). Pelagic fish are phylogenetically far removed from birds and mammals and they move with six degrees of freedom through three-dimensional space, so are a natural vertebrate group to test the generality of this hypothesis. If the hypothesis holds, then we would expect that fish should show no difference in accuracy in encoding spatial information in the horizontal or the vertical dimensions of space. Here, we summarize a number of recent behavioral experiments that begin to unpick the mechanisms that are used by fish to navigate through three-dimensions. In these experiments, we control the inputs to an animal while observing the outputs. By doing so, we investigate the way that fish encode three-dimensional space and we make inferences about the underlying processing.

By training banded tetra fish (*Astyanax fasciatus*) through a rotatable Y-maze in an operant conditioning paradigm (Figure 1), we showed firstly that pelagic fish are able to separate the vertical and horizontal dimensions, and secondly, that they have a preference for the vertical when the previously learned vertical and horizontal components conflicted. This result holds both in the presence and absence of visual landmarks (Holbrook and Burt de Perera, 2009, 2011b). Further, fish were able to extract the horizontal component from a learned three-dimensional path, revealing that they had learned and remembered spatial information from both dimensions. Similar results were also found with benthic fish (*Corydoras aeneus*), which also showed a preference for the vertical component (Davis et al., 2014). This strong vertical preference might be due to the presence of an extra cue in the vertical dimension: hydrostatic pressure, which is a global, stable cue that could allow a fish to ascertain its depth (Taylor et al., 2010; Holbrook and Burt de Perera, 2011a).

**Figure 1:**
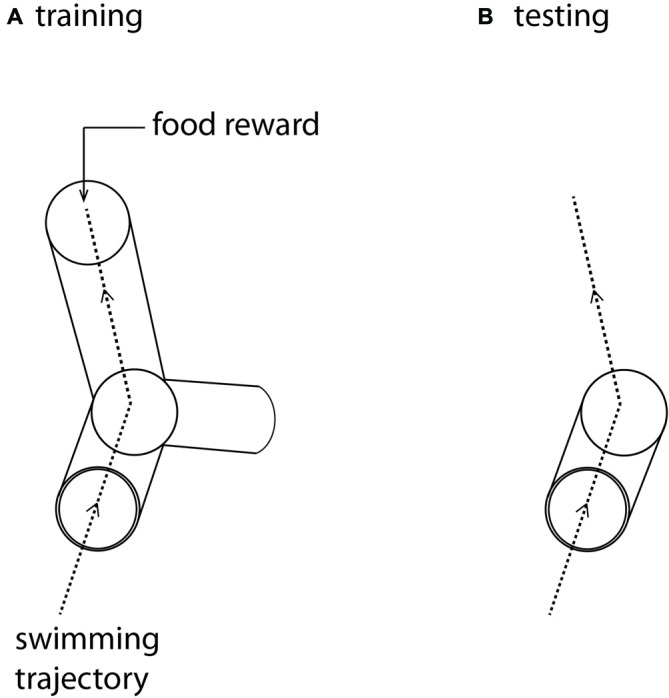
**(A)** The training maze. The three-dimensional Y-maze was made from clear Perspex and supported on a clear Perspex stand, enabling it to rotate around its axis. The maze was positioned in the center of a large cuboid tank (0.58 × 0.58 m and 0.56 m). Fish were placed in a start box at the front of the maze and given a rest period of 1 min before the start box door was removed, allowing them to swim into the Y-maze. Fish were trained to swim towards a food reward placed at the end of one of the arms, oriented so that the fish were trained to swim either forward, up and left or forward, down and right. **(B)** The test maze was identical to the first part of the training maze, but the arms were removed. After training in **(A)** fish were again placed in a start box at the front of this open maze. In test trials they were allowed to swim freely through the whole volume of the cuboid aquarium. The dotted lines represent the trajectory that trained fish swim in this particular trial **(A)**, and the expected trajectory under test conditions **(B)**.

As with hummingbirds, the vertical component of space is important to fish. But is there a difference in the accuracy of memory between the vertical and horizontal components? In a similar set up to the above, we again trained individual *A. fasciatus* fish to swim through a three-dimensional Y-maze placed within a glass tank (Holbrook and Burt de Perera, 2013). In test trials the arms of the maze were removed so that the fish could swim freely on exiting the horizontal section of the maze (Figure 1). The three-dimensional trajectories of individual fish in the final training trial and the test trials were recorded at high spatial and temporal resolution (25 Hz). We then calculated the fidelity of these trajectories relative to the final training trajectory for: (a) just the horizontal component of the data; (b) just the vertical component; and (c) the three-dimensional data. During the test trials, fish reproduced the three-dimensional training trajectories with remarkable accuracy. We hypothesized that fish, unlike rats, might represent horizontal and vertical space equally. In other words, we predicted that there would be no significant difference in error in the behavioral output (swimming trajectories) between the horizontal and vertical dimensions. Our results matched this expectation—there was no difference in error between the two dimensions in terms of fidelity (Burt de Perera and Holbrook, 2012; Holbrook and Burt de Perera, 2013). Further analyses of the trajectory data also reveals that the efficiency of the free-swimming path, as defined by the shortest path to the reward, was similar in the horizontal and vertical dimensions (paired *t*-test: *t*_8_ = 0.76, *P* = 0.46, Figure 2).

**Figure 2:**
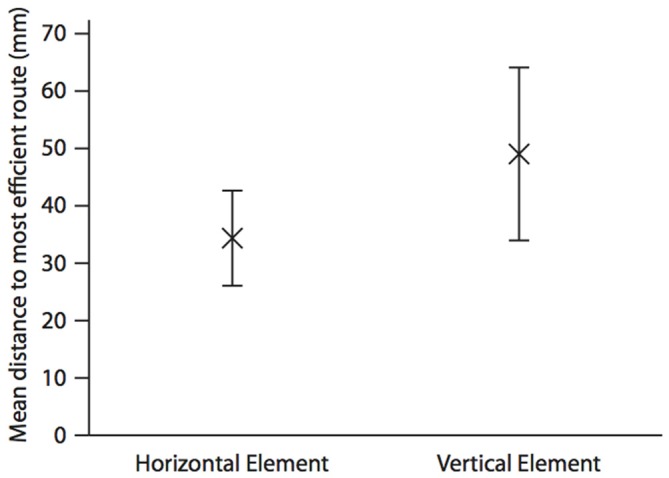
**Mean (±SEM) distance (mm) in the horizontal and the vertical elements of the test trajectory to the most efficient route to the reward.** The most efficient route is defined as a straight line from the position the fish leaves the horizontal element of the Y-maze to the food position during the last training trial. This is calculated by taking each point along the test trajectory and calculating the shortest distance between this and the most efficient route using either only the horizontal, and only the vertical parts of the three-dimensional Cartesian coordinate. Analysis is completed until the fish moves towards, rather than away from, the point at which it left the horizontal portion of the maze in the test trial. The mean is calculated for the whole trajectory. *N* = 9; data from Holbrook and Burt de Perera (2013).

This similarity in error between the horizontal and vertical components of a route suggests that these two components of space are represented in the brain with equal accuracy. Given our initial results (that when put into conflict, information in the vertical axis is preferred over the horizontal dimension) this is intriguing. One possibility is that spatial information from both horizontal and vertical dimensions is processed upstream, where it is finally stored in a fully three-dimensional representation of space. This could take the form of the piscine equivalent of place fields, and through ontogeny or evolution have a particular spherical form rather than a prolate spheroid as seen in rats (Hayman et al., 2011).

There are three possible mechanisms by which the horizontal and vertical error may not differ in our experiments: the motor output may be equivalent between the dimensions; the fish use the same sensory input in both dimensions; or sensory information might be integrated and metric information processed before being encoded isotropically in a higher order location in the brain.

Firstly, a learned ideomotor response might control the direction of swimming. However, this cannot be the entire solution. Dependence on a learned ideomotor response is that it is highly susceptible to turbulence and flow and would be of limited use in most aquatic environments. Even a small amount of turbulence in the test arena (which would be small compared to the wild) would make this mechanism unreliable. In addition to this, fish exited the maze from different locations in test compared to the training trials, yet some still replicated the training path. Our research has shown that fish learning a route to a reward that comprises both vertical and horizontal information show a preference for the vertical when the two are in conflict, yet are able to extract the learned horizontal information (Holbrook and Burt de Perera, 2009). These results would be impossible if the swimming movements were not under cognitive control. If this behavior were solely controlled by motor responses this would result in these fish not leaving the maze, or possibly randomly choosing to swim either left or right.

A second possible explanation is that information is obtained from the same sensory input, with no subsequent modulation. In other words, the fish receive one stream of information that contains both horizontal and vertical cues, and that is not separated into different components that are tied to each dimension. This is unlikely, given the cues that are available to the fish to navigate through three-dimensional space, and indeed it has been shown that fish separate the vertical and horizontal dimensions of space during a navigation task (Holbrook and Burt de Perera, 2009). In the vertical axis, the fish might have used hydrostatic pressure to navigate (Taylor et al., 2010; Holbrook and Burt de Perera, 2011a), or they might have used proprioceptive information to inform turning direction—cues which are known to be used by fish to navigate horizontally (Sutherland et al., 2009). In our experiments (e.g., Holbrook and Burt de Perera, 2013), most sensory cues were rendered uninformative (for example, olfactory cues). Therefore the most likely cues that could have been used in both horizontal and vertical dimensions at the same time were proprioceptive. However, these must be different in the horizontal and vertical given the different swimming modes in subcarangiform fishes. On exiting the start of the maze, the fish are forced to swim directly forwards. To swim up or down, the fish would need to change the orientation of their paired fins to change their pitch. To change body orientation in the horizontal plane to turn left or right the fish could simply introduce an asymmetry into the tail beat or their pectoral fins. From this we surmise that the fish cannot be obtaining information from the same sensory input to navigate in three-dimensions.

The final, and we argue most likely, explanation for the equal error between the horizontal and vertical dimensions of space is that fish have a supramodal (combining information from many sensory modalities) metric representation of three-dimensional space that is equivalent in the horizontal and vertical components. Rats process sensory information upstream of the hippocampus, where metric information is extracted and routed via head direction and grid cells. This metric information is integrated to produce coherent firing fields in populations of place cells within the hippocampus (Jeffery, 2007). We hypothesize that fish process sensory information in a similar way, by integrating information from the senses, before feeding it into a population of neural cells that lie downstream. If these supramodal neural cells exist in fish, our expectation is that they will have roughly spherical activity fields, similar to bats, which would also be consistent with the equivalence in navigational error in the horizontal and vertical dimensions. A possible location for these cells would be in the lateral pallium, which lesion studies have shown to be the functional equivalent of the mammalian hippocampus (Rodríguez et al., 2002; Broglio et al., 2011).

Together, the evidence points to fish using similar mechanisms to encode space as other vertebrates. However, differences in the errors between vertical and horizontal spatial memory in surface bound rats on one hand, and freely-moving bats, birds and fish on the other (both in behavioral output, and mechanistically, in the shape of firing fields of the neural cells that encode space), suggests that the accuracy of three-dimensional spatial representation is defined by the animals’ freedom of movement.

There is, however, a problem in making inferences from separate neurophysiological and behavioral evidence. The neural studies cannot measure accuracy of spatial encoding in the vertical dimension, but can only quantify precision. In rats, the place fields are stretched vertically in relation to the horizontal, which indicates that the fields are less well resolved in the vertical dimension (Hayman et al., 2011). While accuracy of navigation might be implied from this, it has not been formally tested. It is only possible to test accuracy using behavioral paradigms, but without simultaneous neural recordings, the underlying processing can only be inferred. To fully understand how spatial information is encoded, and to ascertain the eventual output of this neural representation, future work will need to integrate these two approaches.

## Author Contributions

All authors discussed the intellectual content of the article, contributed to its design and contributed text. TBdP collated/edited the text and wrote the bulk of the article.

## Conflict of Interest Statement

The authors declare that the research was conducted in the absence of any commercial or financial relationships that could be construed as a potential conflict of interest.
